# Metagenomic Insights into Chicken Gut Antibiotic Resistomes and Microbiomes

**DOI:** 10.1128/spectrum.01907-21

**Published:** 2022-03-01

**Authors:** Jintao Yang, Cuihong Tong, Danyu Xiao, Longfei Xie, Ruonan Zhao, Zhipeng Huo, Ziyun Tang, Jie Hao, Zhenling Zeng, Wenguang Xiong

**Affiliations:** a Guangdong Provincial Key Laboratory of Veterinary Pharmaceutics Development and Safety Evaluation, College of Veterinary Medicine, South China Agricultural University, Guangzhou, China; b Guangdong Laboratory for Lingnan Modern Agriculture, Guangzhou, China; c National Laboratory of Safety Evaluation (Environmental Assessment) of Veterinary Drugs, College of Veterinary Medicine, South China Agricultural University, Guangzhou, China; d National Risk Assessment Laboratory for Antimicrobial Resistance of Animal Original Bacteria, College of Veterinary Medicine, South China Agricultural University, Guangzhou, China; Universidad de Buenos Aires, Facultad de Farmacia y Bioquímica

**Keywords:** antibiotic resistance genes, metagenome, chicken gut, host-tracking, mobile genetic elements

## Abstract

The chicken gut microbiota, as a reservoir of antibiotic resistance genes (ARGs), poses a high risk to humans and animals worldwide. Yet a comprehensive exploration of the chicken gut antibiotic resistomes remains incomplete. In this study, we established the largest chicken gut resistance gene catalogue to date through metagenomic analysis of 629 chicken gut samples. We found significantly higher abundance of ARGs in the Chinese chicken gut than that in the Europe. *tetX*, *mcr*, and *bla*_NDM,_ the genes resistant to antibiotics of last resort for human and animal health, were detected in the Chinese chicken gut. The abundance of ARGs was linearly correlated with that of mobile genetic elements (MGEs). The host-tracking analysis identified Escherichia, *Enterococcus*, Staphylococcus, Klebsiella, and *Lactobacillus* as the major ARG hosts. Especially, *Lactobacillus*, an intestinal probiotic, carried multiple drug resistance genes, and was proportional to IS*Lhe63*, highlighting its potential risk in agricultural production processes. We first established a reference gene catalogue of chicken gut antibiotic resistomes. Our study helps to improve the knowledge and understanding of chicken antibiotic resistomes for knowledge-based sustainable chicken meat production.

**IMPORTANCE** The prevalence of antibiotic resistance genes in the chicken gut environment poses a serious threat to human health; however, we lack a comprehensive exploration of antibiotic resistomes and microbiomes in the chicken gut environment. The results of this study demonstrate the diversity and abundance of antibiotic resistance genes and flora in the chicken gut environment and identify a variety of potential hosts carrying antibiotic resistance genes. Further analysis showed that mobile genetic elements were linearly correlated with antibiotic resistance genes abundance, implying that we should pay attention to the role played by mobile genetic elements in antibiotic resistance genes transmission. We established a reference genome of gut antibiotic resistance genes in chickens, which will help to rationalize the use of drugs in poultry farming.

## INTRODUCTION

Antibiotic resistance has been recognized as a significant global threat to public health and food safety by the World Health Organization (1). Currently, hundreds of thousands of people die each year from infections caused by antibiotic-resistant bacteria. Irrational use of antibiotics is thought to be the main driver for the development of antibiotic resistance (2). Antibiotic resistance genes (ARGs) are widely distributed in surface water, sewage treatment plant effluent, soil, and animal feces (3). In livestock farming environments, ARGs and drug-resistant bacteria can be transmitted to humans through the food chain, water, or air. Besides, the horizontal gene transfer (HGT) process makes it possible for bacteria from various ecological niches to share genes, thereby playing a vital role in the dissemination and evolution of ARGs. Undoubtedly, the gut atmosphere offers a strong communication site. A significant number of ARGs have been identified in the poultry gut (4).

As the world's population grows, the demand for animal protein including poultry meat, as a high-quality protein, is increasing day by day. Broiler chickens are considered one of the world's main meat suppliers. Global poultry meat consumption increased from 11 kg per capita in 2000 to 14,4 kg per capita in 2011 and is projected to reach 17,2 kg per capita in 2030 (5). The production of poultry meat will reach 134.5 million tons in 2023, according to a report submitted by the Organization for Economic Cooperation and Development and the Food and Agriculture Organization (6). The modernization of livestock farming is inseparable from the use of antibiotics, and it is estimated that the global consumption of antimicrobials will increase by 67% between 2010 and 2030 (7). The continued use of antibiotics would alter the microbial structure of the animal gut and cause an increase in abundance of antibiotic resistance genes in the animal gut. This anthropogenic activity promotes the emergence and spread of antibiotic resistance genes including *tetX*, *mcr*, and *bla*_NDM_, the genes resistant to antibiotics of last resort for human and animal health.

Tigecycline is one of the last resorts for the treatment of complex infections caused by multi-resistant Gram-negative and Gram-positive bacteria. In 2019, two variants of plasmid-encoded *tetX*, named *tetX3* and *tetX4*, were identified in Enterobacter and Acinetobacter isolated from animal and human sources. Plasmids carrying *tetX3* and *tetX4* are already widely distributed in human pathogens and are transferable and stable (8). Polymyxins are important lipopeptide antibiotic that serves as the last line of defense against multidrug-resistant Gram-negative bacterial infections (9). In November 2015, the first plasmid-mediated polymyxin resistance gene, *mcr-1*, was discovered in China (10). *mcr-1* and its variants have been identified in more than 50 countries to date. The *mcr-1* gene is carried by multiple plasmids, including IncX4, IncHI2, and IncI2, which strengthens the risk of further *mcr-1* transmission worldwide. The *bla*_NDM_ gene encodes a carbapenemase that hydrolyzes almost all β-lactam antibiotics. Carbapenem-resistant *Enterobacteriaceae* that produce the *bla*_NDM_ gene are found worldwide and have become a major public health threat.

Due to the diversity and abundance of microorganisms in the gut microecology, the human and animal gut is an important site for the increase and spread of ARGs, with a large number of ARGs settling and spreading to the gut flora through host cells. Currently, studies of gut resistance groups have been conducted in a variety of animals, such as humans, pigs, and chickens (11), which have deepened the understanding of the distribution, evolution, and transmission of ARGs in the gut flora. However, in general, there is still no standard process for metagenomic analysis.

In this study, we explored the comprehensive profile of ARGs in chicken gut by using large-scale metagenomic data sets. We established the largest chicken gut resistance gene catalogue to date through metagenomic analysis of 629 chicken gut samples. We analyzed the diversity, abundance of ARGs and ARG hosts from multiple perspectives. Besides, we reconstructed 4062 high-quality metagenome-assembled genomes (MAGs) using a binning approach, most of which belong to species lacking culture representation. Combining these individual efforts and establishing a unified non-redundant data set of the chicken gut genome is essential to advance future microbiome studies. We think these data will lay the foundation for future studies on the structure of chicken gut microbiota.

## RESULTS

### Broad-spectrum profile of ARG abundance.

In total, 387ARG subtypes belonging to 14 ARG types were detected in at least one of the 629 chicken gut samples, with total abundances ranging from 0.031 to 3.42 × 10 copies/cell (Table S3). The resistance genes to tetracycline (34.24%), aminoglycoside (19.37%), macrolide (19.37%), phenicol (10.94%), sulfonamide (5.46%), beta-lactam (5.09%), oxazolidinone (1.88%), quinolone (1.70%), were more abundant and prevalent in these samples.

**FIG 1:**
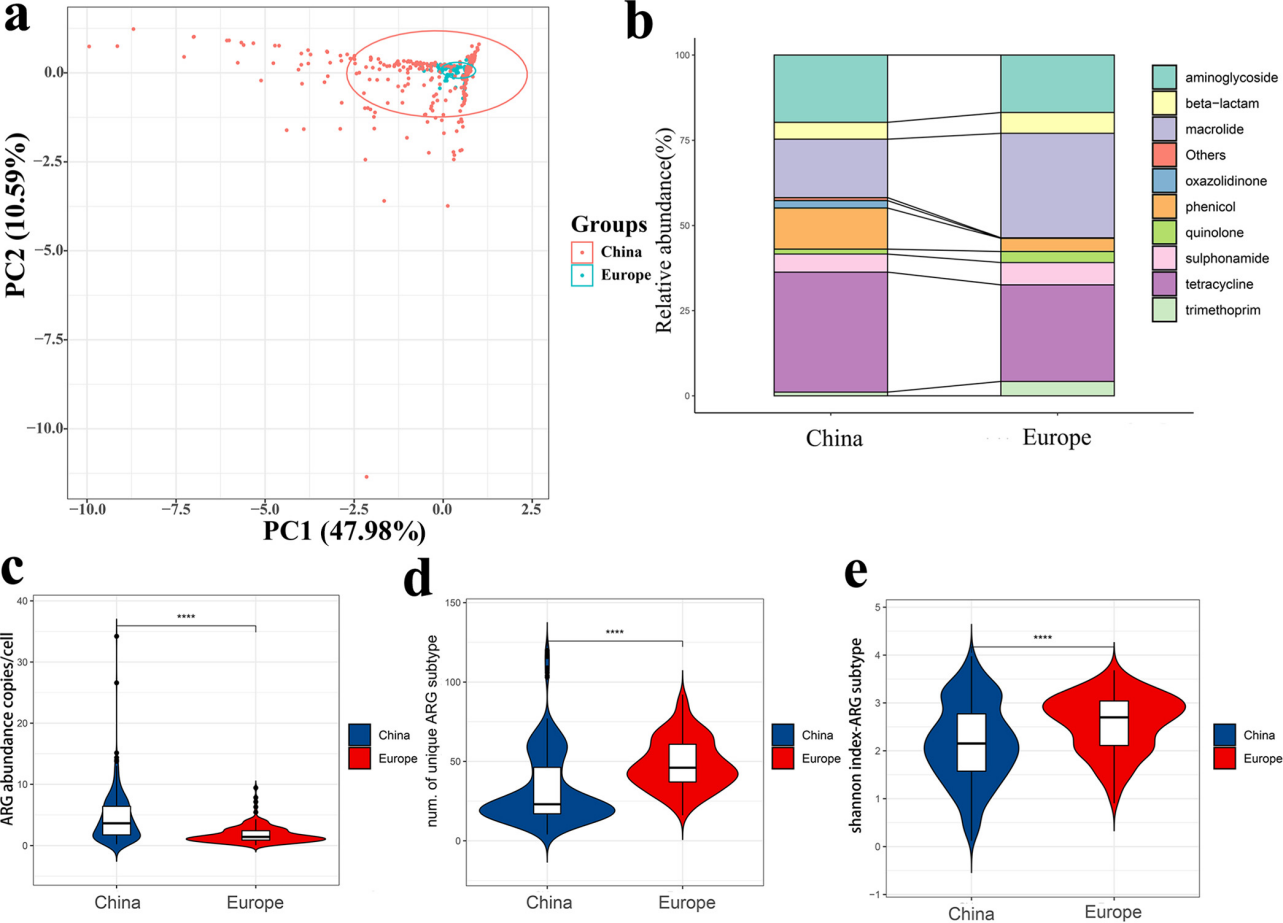
ARG composition structure distribution. (a) PCA plots showing the ARG subtypes composition differences among the 618 chicken gut samples. (b) Comparison of ARG types in China and Europe. (c) ARGs abundance comparison. (d) Diversity of ARGs subtypes. (e) ARGs subtype of Shannon's Diversity Index.

Depending on the geographical location of the samples, we could divide them into two groups, China and Europe (due to the small number of samples in the USA, there were no group discussions). Overall, the average abundance of ARG types in the chicken gut was 3.62 copies/cell. The tetracycline (1.24 copies/cell), aminoglycoside (0.70 copies/cell), and macrolide (0.69 copies/cell) resistance genes were the predominant ARG types. (Fig. 1b). The abundance of ARG types in China was significantly different from that in Europe (*P < *0.0001). The average abundance of ARGs was about three times higher in China than that in Europe (Fig. 1c).

The similarity of ARG subtypes compositions was calculated using principal-component analysis (PCA). Chicken gut samples were well clustered in Europe, whereas in China they were more dispersed, suggesting that the Chinese region has a more diverse ARG composition than that in Europe (Fig. 1a). Each sample carried a different number of species of ARGs, so we further compared the diversity of ARGs between China and Europe. Interestingly, the results showed the presence of more diverse resistance gene types in Europe (Fig. 1d and e). This may be related to the fact that the samples collected in Europe were spread over nine countries.

Of the 387 ARG subtypes detected in the chicken gut, the top five abundant ARG subtypes were considered representative ARGs, *tetW*, *ermB*, *tetA*, *tetL*, and *sul2.* Interestingly, we found that more than half of these five dominant ARG subtypes were tetracycline resistance genes, of which *tetW* accounted for 18.25% of the overall.

**FIG 2:**
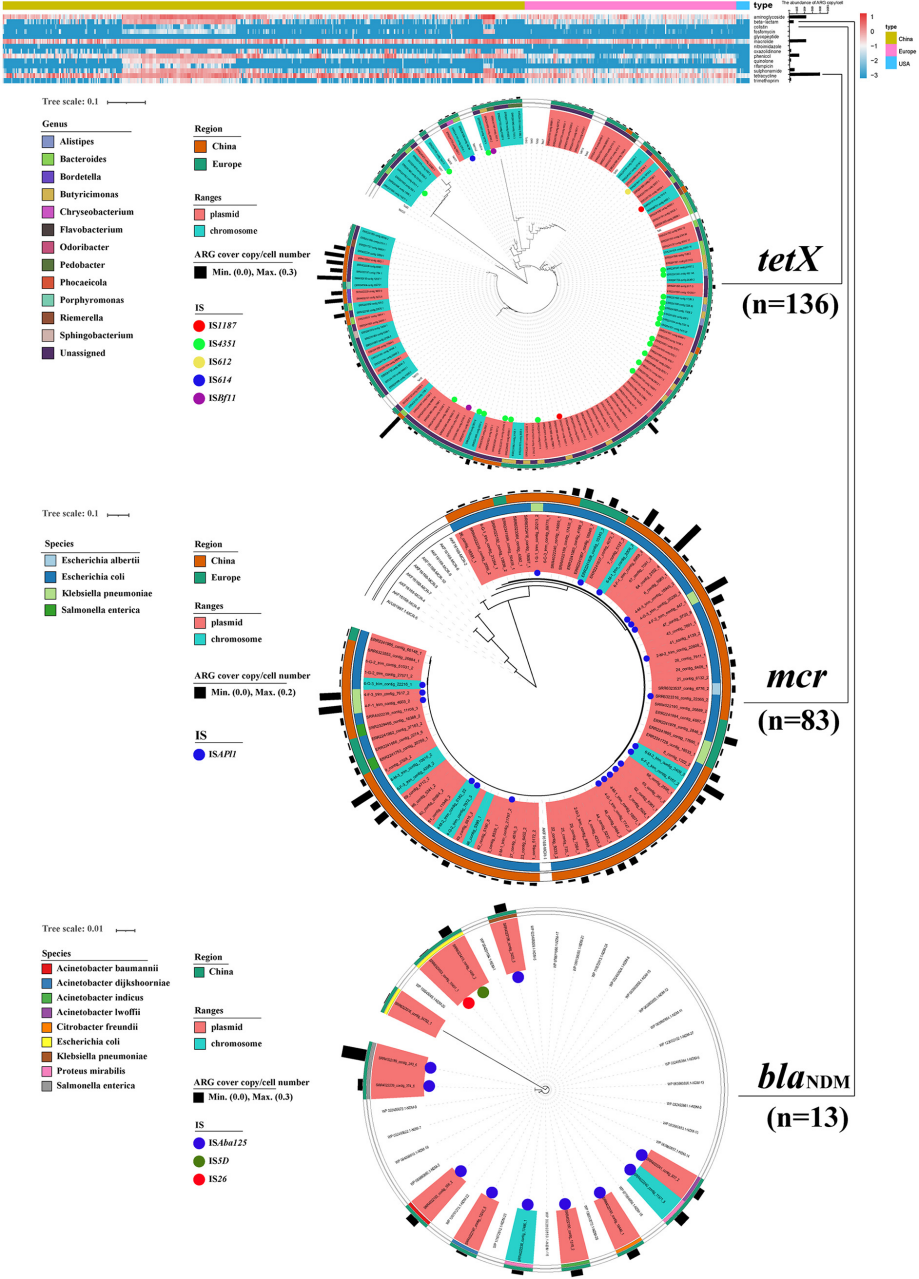
ARGs heatmap and phylogenetic tree of *tetX*, *mcr*, and *bla*_NDM_ detected in the chicken gut.

The *tetX*, *mcr*, and *bla*_NDM_ were all detected to varying degrees in 629 chicken gut samples. The *tetX* was detected more frequently in European chicken gut samples (93 of 178, 3.78 × 10^−4^ −1.18 × 10^−1^ copies/cell) than that in Chinese (17 of 440, 6.93 × 10^−3^ −3.67 × 10^−1^ copies/cell). Besides, the *mcr* was detected more frequently in Chinese chicken gut samples (70 of 440, 1.61 × 10^−3^ −1.63 × 10^−1^ copies/cell) and to a lesser extent in European chicken gut samples (13 of 178, 1.23 × 10^−3^ −5.99 × 10^−2^ copies/cell). Also, we found that *bla*_NDM_ was only detected in Chinese chicken gut samples (11 of 440, 2.79 × 10^−3^ −2.51 × 10^−1^ copies/cell). Furthermore, we obtained further distributions by constructing a phylogenetic tree analysis of these genes. Concerning *tetX* genes, *tetX10* and *tetX2* were more prevalent in the chicken gut and most were present on plasmids. Most of *tetX* belonged to the *Bacteroides* and *Butyricimonas*. IS*4351*, IS*1187*, IS*612*, IS*614*, and IS*Bfl1* may have an important role in the transfer of *tetX*. Most of *mcr-1* were present on plasmids that were attributed to Escherichia coli, Escherichia albertii, and Klebsiella pneumonia. As well, IS*Apl1* was the predominant transposon for *mcr-1* transfer. Most of *bla*_NDM_ were also present on plasmids carried by a variety of pathogenic bacteria such as Escherichia coli, Salmonella enterica, and Proteus mirabilis. IS*Aba125*, IS*5D*, and IS*26* were potential for their transfer vectors (Fig. 2).

### Bacterial community structure.

**FIG 3:**
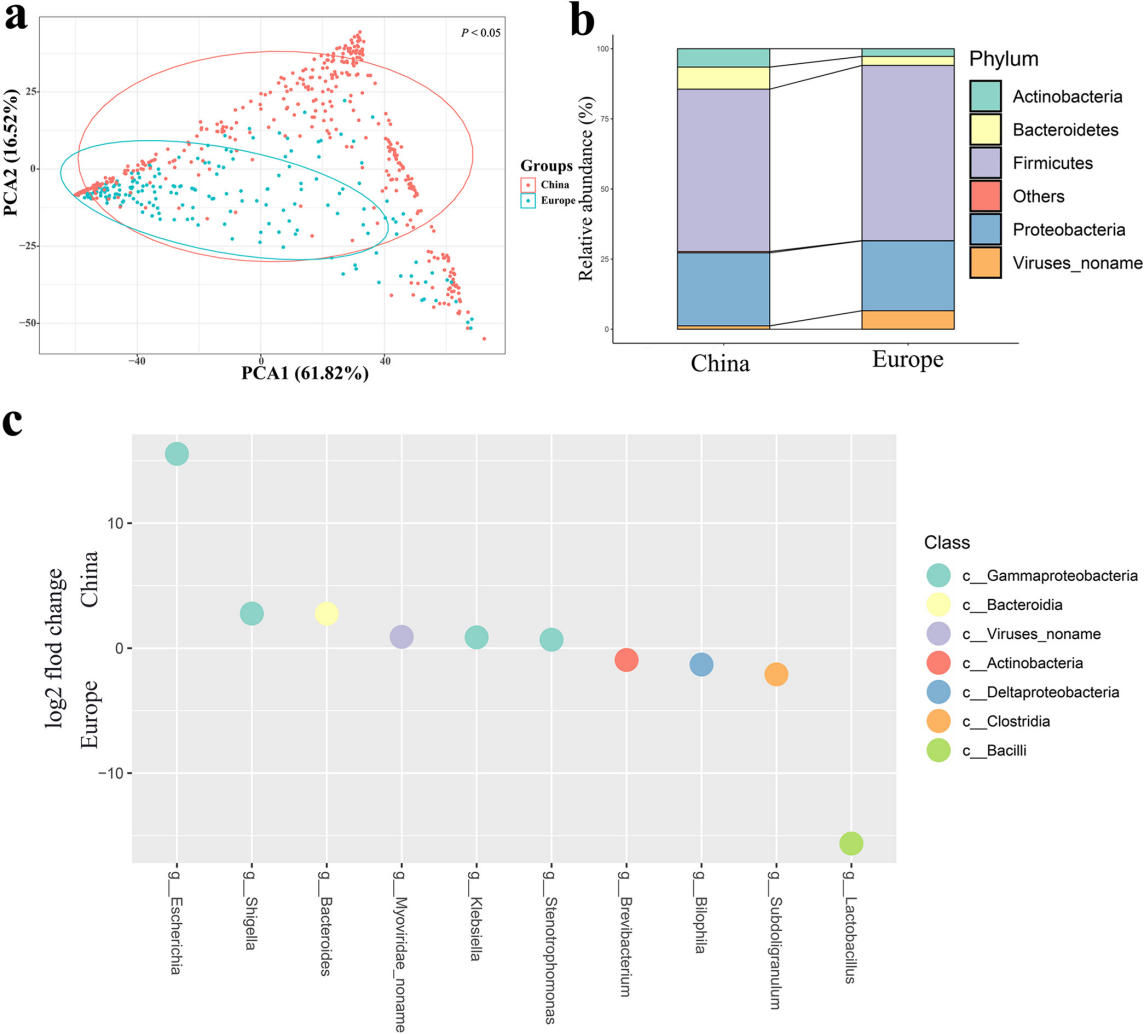
Bacterial population structure distribution. (a) PCA plots showing the Bacterial flora (genus level) composition differences among the 618 chicken gut samples. (b) Comparison of bacterial flora in China and Europe. (c) Differentially abundant bacteria. Differences in genus abundance between China and Europe. The *y* axis represents the log_2_ fold change and the *x* axis represents the genus name.

Overall, the *Firmicutes* (59.13%), *Proteobacteria* (25.38%), *Bacteroidetes* (7.05%), *Actinobacteria* (5.36%) were the predominant phyla in the chicken gut samples. While there was no variation between the Chinese and European samples in the dominant bacterial phylum (Fig. 3b). *Lactobacillus* and Escherichia had the highest abundance percentages of 42.47% and 18.43%, respectively. It was hypothesized that the changes in the abundance of *Lactobacillus* and Escherichia in the chicken gut were the main reasons for the changes in the percentages of their corresponding phyla. Interestingly, we find that the European samples cluster together, while the Chinese samples wrap around the European samples (Fig. 3b), which is highly similar to the previous ARG subtypes compositions. *Lactobacillus* and Escherichia were the two genera with the most significant differences between the Chinese and European groups, respectively (Fig. 3c). The correlation analysis revealed that the abundance of *Lactobacillus* and Escherichia were negatively correlated (ρ = −0.503, *P < *0.01, Spearman's correlation coefficient, two-tailed test).

Previous studies have shown that bacterial community structure determines the distribution of resistance genes without considering HGT, and a significant correlation between bacterial composition and ARG profiles could observe (12, 13). Visualized by the Procrustes analyses, ARGs and bacterial composition in the chicken gut showed highly significant goodness of fit by ANOVA (M^2^ = 0.7888, *P < *0.001) (Fig. S1).

### The host of ARGs in chicken gut samples.

After filtering the contigs with lengths less than 500 bp, we obtained a total of 35672711 contigs from 629 samples, of which 24318 contigs were identified as ARG-carrying contigs (ACCs) (Table S2). Only 10809 (44.5%) of these contigs could be annotated to the genus level (244 genera), suggesting that we have limited knowledge of the hosts carrying ARGs.

**FIG 4:**
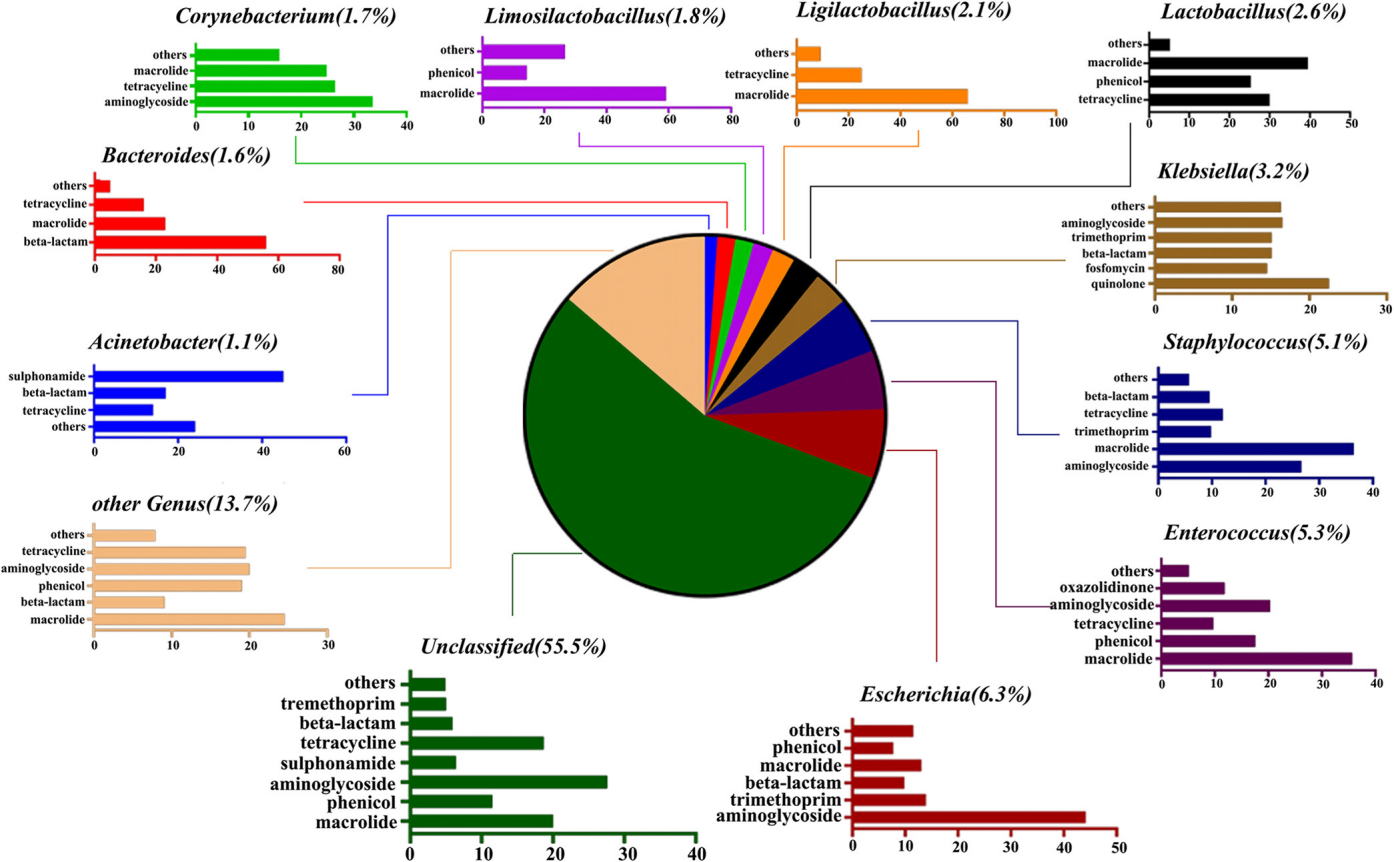
The taxonomy of ACCs (in genus level) and the percentages of ARG types these contigs carried. For example, Escherichia (6.3%) represents that 6.3% of ACCs were annotated as Escherichia. Bar chart shows the percentages of ARG types that were carried by the annotated ACCs. For example, 44.1% of the ARG-carrying contigs originating from Escherichia carried aminoglycoside resistance genes.

Overall, at the phylum level, *Firmicutes* (42.6%), *Proteobacteria* (28.7%), and *Bacteroidetes* (4.8%) were the major ARG carriers (Table S4). At the genus level, Escherichia (6.3%), *Enterococcus* (5.3%), Staphylococcus (5.1%), Klebsiella (3.2%), *Lactobacillus* (2.6%), *Ligilactobacillus* (2.1%), *Limosilactobacillus* (1.8%), *Corynebacterium* (1.7%), *Bacteroides* (1.6%), and Acinetobacter (1.1%) were the main ARGs hosts (Fig. 4). Besides, we found that the dominant genus Acinetobacter carries the highest percentage of sulfonamide resistance genes (53.78%). Wang et al. also identified a novel strain capable of degrading sulfamethoxazole in 2018, identified as Acinetobacter *sp* (14). Escherichia was the most common carrier of ARGs. Escherichia had a high percentage of 94.00% for colistin resistance genes, in addition to the highest percentage for aminoglycosides and trimethoprim resistance genes with 28.89% and 33.07%, respectively. All glycopeptide resistance genes were found in *Enterococcus*, while *Enterococcus* had the highest percentage of resistance genes for macrolides, oxazolidinones, and chloramphenicol with 15.25%, 94.44%, and 16.35%, respectively. Klebsiella was also found to have the highest proportion of resistance genes in phosphomycin, quinolone, and rifampicin with 52.36%, 67.84%, and 93.18%, respectively. *Bacteroides.* had the highest percentage of β-lactam and nitroimidazole resistance genes (23.04% and 73.33%, respectively). *Lactobacillus*, as a probiotic, was also the genus that accounted for more of the macrolide and tetracycline, accounting for 8.19% and 12.27%, respectively (Table S5). The predominant genera of *Bacteroides*, Escherichia, Klebsiella, and Staphylococcus that carry ARGs are mostly considered as conditional pathogens (15), and their ability to colonize the chicken gut may increase the exposure and transmission of ARGs.

### Mobile genetic elements.

To better evaluate the potential transferability of ARGs in chicken gut samples, we tracked the ARGs associated with MGEs. Overall, the average abundance of MGEs in the chicken gut was 8.01 copies/cell. We identified a total of 10971 contigs carrying integrase sequences, 19830 contigs carrying insertion sequences, and 8589 contigs carrying plasmid replicons, which have great HGT potential. Meanwhile, we further screened the ARGs that co-occurred with MGEs (ARGs have at least one MGE next to them on the contig) that have been identified for mobilized ARGs (16), and we found 6409 (29.91%) contigs with coexisting ARGs and MGEs out of 21431 ACCs and showed the distribution of different types of MGEs among their 6409 contigs (Fig. S2).

The phylum with the highest percentage of these 6409 contigs with coexisting ARGs and MGEs was *Proteobacteria* (62.16%), while at the level of the genus, the highest percentage was Escherichia (12.65%), followed by Klebsiella (6.54%). Also, in terms of the types of drug resistance included, the highest percentage was aminoglycosides (26.56%), followed by phenicol, β-lactam, and sulfonamide with 16.54%, 13.90%, and 13.48%, respectively. We further explored the correlation between *intI1* and ARGs. *intI1* was found to be correlated with *aph(6)-Id*, *bla*_OXA-10_, *bla*_TEM-1B_, *mphA*, *floR*, *qnrS1*, *sul1*, *sul2*, *sul3*, and *tetA*, which are drug resistance gene subtypes significant positive correlations (Table S6).

**FIG 5:**
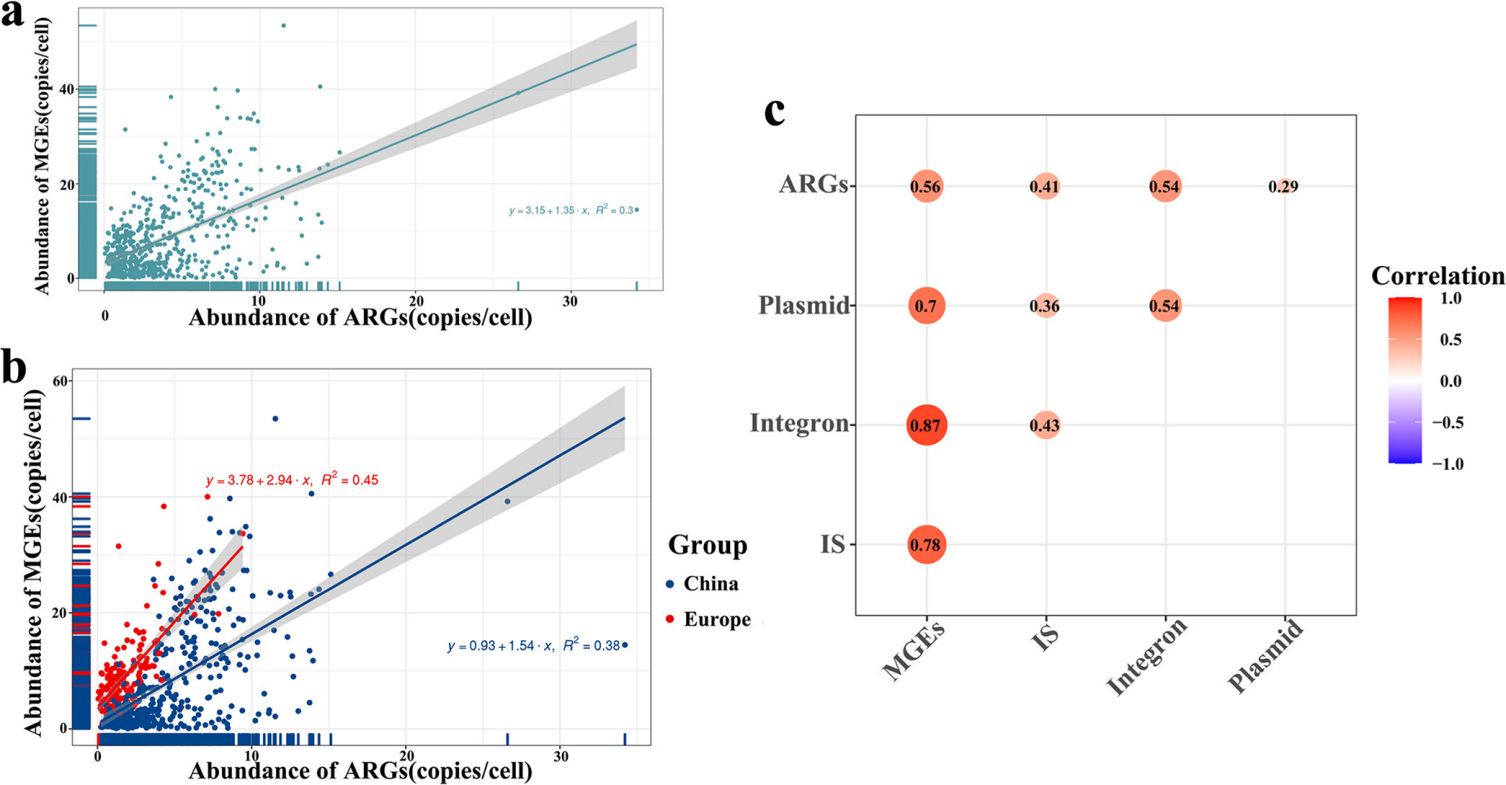
Correlation between ARGs and MGEs. (a) Overall correlation of ARGs abundance and MGEs abundance in 629 chicken gut samples. (b) Correlation of ARGs abundance and MGEs abundance by Chinese and European groups. (c) Heatmap of correlation between MGEs, IS, plasmid and integron.

We found a linear correlation between the abundance of MGEs and the abundance of ARGs (y = 3.15 + 1.35 x, R^2^ = 0.3) (Fig. 5a), and also the correlation heat map produced by ggcorrplot package showed a correlation between ARGs and MGEs (*r* = 0.56, *P < *0.05) (Fig. 5c), indicating that the presence of MGEs plays a transfer of ARGs an important role. We found the highest abundance of IS*Lhe63* among the IS and IS*Lhe63* showed a significant correlation with *Lactobacillus* (ρ = 0.621, *P < *0.05), which is consistent with previous studies (17).

Using ARGs structure as the response variable, the top 10 genus in terms of abundance and 3 types of MGEs (Integron, IS, Plasmid) as explanatory variables for RDA, after forward model selection, provided 12 explanatory variables, all of which were significantly positively correlated except for *Bacteroides* (Fig. S3a). VPA was used to determine the co-occurrence of MGEs and genus on ARGs structure. The results showed that the most important influences on the structure of ARGs were MGEs, with individual explanatory rates of 12.1% and 6.6%, respectively (Fig. S3b).

### Metagenome-assembled genomes from the chicken gut.

On each of the 629 samples, we carried out a single-sample metagenomic assembly. A total of 25598 MAGs were created from binning. With a total of 4062 high-quality genomes with completeness ≥ 80% and contamination ≤ 10%, we were left. Of these 1823 had completeness > 90% and contamination < 5%, 938 were > 95% complete with < 5% contamination, and 163 MAGs were > 97% complete with 0% contamination. The distribution of these MAGs between the 629 samples can be found in Table S7.

Table S7 contains the GTDB-TK classification of each high-quality MAGs, and Fig. S4 shows the phylogenetic tree of MAGs, which helps to further correct the occurrence of classification errors. Overall, the most dominant clade was *Firmicutes* (*n* = 2551), followed by *Bacteroidot*a (*n* = 803), *Actinobacteriote* (*n* = 345), *Proteobacteria* (*n* = 176), *Campylobacterota* (*n* = 71), *Desulfobacterota* (*n* = 57), and so on We also identified four archaea, one *UBA71 sp006954425*, two *Methanobrevibacter_A woesei* and one *Methanocorpusculum*.

**FIG 6:**
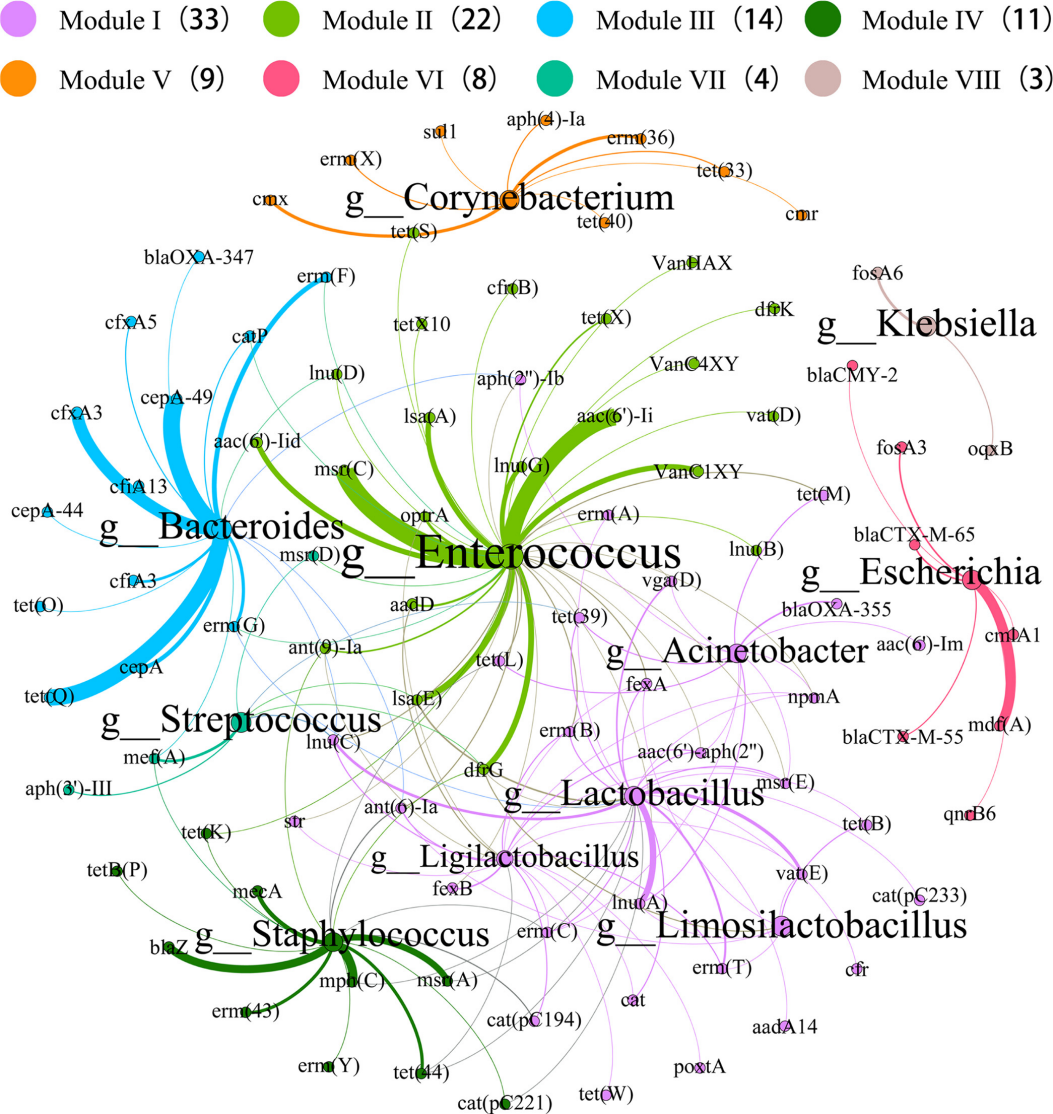
The network analysis revealing the co-occurrence patterns between major MAGs (in genus level) and ARG subtypes. The nodes were colored according to the modularity class. Modularity index 0.68. The size of each node was proportional to the number of connections, that is, the average weighted.

Previous research analyzing the possible hosts of ARGs in metagenomic data usually used the method of correlation network analysis (18), but this solution has the downside that only a small number of ARG hosts can be revealed and further confirmation is required. A recent study has explored potential hosts by ARGs-like-ORFs in assembled contigs (19). However, only partial hosts of ARGs could be revealed and a large number of ARGs were not precisely assigned to genera. Therefore, we explored ARG potential hosts from multiple perspectives by ARGs-like-ORFs in assembled contigs and by using binning to obtain MAGs. We further screened the high-quality MAGs and obtained 655 MAGs carrying ARGs. As shown in Fig. 6, Enterococcus carries various ARGs such as glycopeptides (*vanC1XY*, *vanC4XY* and *vanHAX*), oxazolidinones (*optrA*), and tetracyclines (*tetX* and *tetX10*). Klebsiella carried fosfomycin (*fosA6*) and Escherichia carried β-lactams (*bla*_CTX-M-65_ and *bla*_CTX-M-55_). It was also observed that *Lactobacillus* carried mainly macrolides (*lnuA* and *vatE*, etc.).

Due to the limitations of the taxonomically annotated reference sequences, some MAGs failed to classify to the genus level. This limitation is common in the taxonomic identification of genomes generated from metagenomic data (20). Despite this, this study still revealed that 156 bacteria genera were the hosts of 140 ARG subtypes. Therefore, this study greatly expands the knowledge of the diversity of antibiotic resistance bacterias in the chicken gut.

## DISCUSSION

Our study analyzed antibiotic resistance based on metagenomic tools within 629 chicken gut samples, demonstrating the correlation between ARGs and MGEs and revealing ARG hosts from multiple perspectives. The microbiome in the chicken gut microbiota constitutes a large reservoir of ARGs. Tetracyclines, aminoglycosides, and macrolides were the most commonly observed ARG forms in this study, whereas certain resistance genes are often present in particular habitats, such as urban waste (21), human intestine (22). However, the major ARGs in this study differed from the most frequently occurring bacitracin in drinking water (19), and also from the soil (12), sludge (13). This suggests that ARGs are not randomly distributed in different environments.

We found that the total level of acquired antimicrobials correlates with the overall livestock antimicrobial use. The average ARG abundance in the gut of chickens in China was three times higher than that in Europe. According to a survey report by the World Organization for Animal Health (OIE) in February 2019, Asia, the Far East, and Oceania had the highest average use of antimicrobials in animals in the world, amounting to 296.75 mg/kg, which is 3.74 times higher than in Europe (23). This value is close to the average abundance ratio of ARGs between China and Europe above. The total ban of all antibiotic growth promoters in the EU member states was in 2006 while it in China was in 2020. Different strategies of human antibiotic use in agricultural practices may be responsible for this phenomenon. Human activities (different strategies for antibiotic use in different geographic areas) may be the main driver of the different distribution of ARGs.

ARGs were frequently detected in the chicken gut, such as *tetX*, *mcr*, and *bla*_NDM_, which may be due to the selection posed by prolonged exposure of gut microbes to antibiotics. Although tigecycline was not applied to livestock animal farming, *tetX* was still found in the gut environment of chickens in this study, presumably due to the selection pressure of tetracyclines. The latest appearance from animals and humans of plasmid-mediated high levels of tigecycline resistance genes *tetX3* and *tetX4* has triggered the much broader public alarm (8), rendering it important for robust surveillance of *tetX* genes to be improved. In November 2015, the plasmid-mediated polymyxin resistance gene *mcr-1* was first identified, subsequently, multiple *Enterobacteriaceae* members with different host origins were found carrying the resistance gene in more than 50 countries per continent, suggesting that *mcr-1* was spread globally (24). And it has been demonstrated that the transposon part Tn6330 (IS*APl1*-*mcr-1*-orf-IS*APl1*) can be removed from the plasmid to form an IS*APl1*-*mcr-1*-orf intermediate loop which can be inserted into other IS*APl1*-carrying plasmids (25, 26). Meanwhile, the gut flora can act as a suitable mixing vessel to facilitate the spread of *mcr* through horizontal gene transfer (26, 27). *bla*_NDM_ genes showed a highly variable region upstream of IS*Aba125*, IS*26*, and IS*5D*. The highly conserved *bla*_NDM-5_ genetic surrounding structures [ΔIS*Aba125*-*bla*_NDM-5_-ble MBL-trpT-dsbD-IS*26*] were found in German and Swiss Escherichia coli of both the F-type and IncX3 plasmids (28–30). But for the potential transfer between IS*5D* and *bla*_NDM_ has not been reported. The *tetX*, *mcr*, and *bla*_NDM_ genes in the chicken gut, especially in China, were detected in the present study. This poses a significant risk to the use of drugs in agricultural practices and deserves our attention. Furthermore, the evidence is growing that genes with antibiotic resistance can be transferred from animals to humans (31). During homeotic development, tracking of *tetX*, *mcr*, and *bla*_NDM_ in the gut should be stressed.

The ARGs present in the MGEs undergoes constant horizontal shifts through human induction and environmental changes (32), which is following Darwinian evolutionary theory. In this study, MGEs were also present in 29.91% of ACCs. We found that the relative abundance of MGEs was linearly correlated with that of ARGs. As the key reason for their survival and spread, horizontal transmission of MGEs to a wide variety of bacteria (including pathogens and human commensals) has been established (33). A large amount of ARGs is stored in the gut of chickens, which may undergo horizontal gene transfer *via* MGEs and is most likely to be transmitted to humans by food chain or spread in the environment, such as rivers and soil, *via* the fecal route (34), indicating the potential risk for human health. To improve monitoring of ARGs and MGEs in the gut tract of chickens and proper handling of livestock manure, this needs to be a concern.

We constructed 4062 high-quality MAGs from 629 chicken gut samples, greatly extending the enrichment of previous chicken gut MAGs (20, 35). Reconstruction of microbial genomes using metagenomic assembly has been applied to humans (36, 37), cattle (38), pigs (39), the bank voles (40), soil (41), and ocean (42), but less in the chicken gut. We found several new strains of *Lactobacillus*, including *L.gallinarum*, *L.crispatus*, and *L.johnsonii*. We also identified several new strains of *Bifidobacterium*, including *B. gallinarum*, *B. globosum*, and *B. animalis*. These strains are considered to be potential chicken gut probiotics (43). It has been shown that probiotics can reduce pathogen transfer in the intestinal mucosa by enhancing intestinal barrier integrity and maintaining immune tolerance. At the level of genus, the most significant difference was Escherichia in China and *Lactobacillus* in Europe. It has been shown that probiotics (especially *Lactobacillus*) have preventive and protective effects against Salmonella enterica and Escherichia *coil O78:K80* infections in chickens (44, 45). Although a variety of *Lactobacillus* is generally considered nonpathogenic for use in a variety of human and animal foods and products, they have a tremendous role in maintaining gut health, such as the bioactive Lactobacillus casei competitively reducing avian gut bacterial pathogens (46). However, it has also been shown that multiple ARGs are isolated from *Lactobacillus* strains and some of them may even transfer their intrinsic ARGs to other lactic acid bacteria or pathogens through horizontal gene transfer (47–50). In our study, *Lactobacillus* was also found to carry a variety of ARGs, especially macrolides and tetracyclines. This suggests that *Lactobacillus* can potentially act as a reservoir of ARGs and may play an active role in the transfer of ARGs to humans through the food chain. We identified 4 archaea in the 4062 high-quality MAGs. It has been shown that *Methanobrevibacter* and *Methanocorpusculum* were positively correlated with all of the detected ARGs (51). Although we did not identify any ARGs in the 4 archaeal strains, it still provides a new idea for a potential link between archaea and ARGs.

## CONCLUSION

We established the largest chicken gut resistance gene catalogue to date through metagenomic analysis of 629 chicken gut samples and revealed the diversity and abundance of ARGs in the chicken gut. The results explained that the differences in the abundance levels of ARGs (e.g., tetX, mcr, and blaNDM) between different regions in China and Europe were completely positively correlated with anthropogenic factors. The widespread presence of MGEs further drives the spread of ARGs through horizontal transmission. Lactobacillus, as gut probiotics, also carried multiple drug resistance genes (especially macrolides and tetracyclines), emphasizing their potential risk. Besides, the number of chicken gut microbial genomes in public databases was enriched by reconstructing a large number of MAGs, but a whole-genome sequencing approach after isolation and culture is still needed to validate the description. Our study helps to improve the knowledge and understanding of chicken gut flora and promote knowledge-based sustainable chicken production.

## MATERIALS AND METHODS

### Metagenomic sample collection.

The 36 gut samples of chicken were collected from our previous study (52). There were 60 chicken gut samples from the current laboratory study. A total of 533 chicken gut samples from 11 countries were randomly selected from NCBI, including China (*n* = 344), Europe (*n* = 178), including Netherlands (*n* = 20), Germany (*n* = 19), France (*n* = 20), Spain (*n* = 20), Belgium (*n* = 20), Italy (*n* = 20), Poland (*n* = 20), Denmark (*n* = 20) and Bulgaria (*n* = 19) and USA (*n* = 11). Furthermore, all the above metagenomic data sets that were sequenced using the Illumina HiSeq platform with PE150 (paired-end sequencing 150 × 2) were downloaded to avoid the fluctuations caused by different sequencing strategies. Additional sample descriptions can be found in Supporting Information Fig. 7 and Table S1.

**FIG 7:**
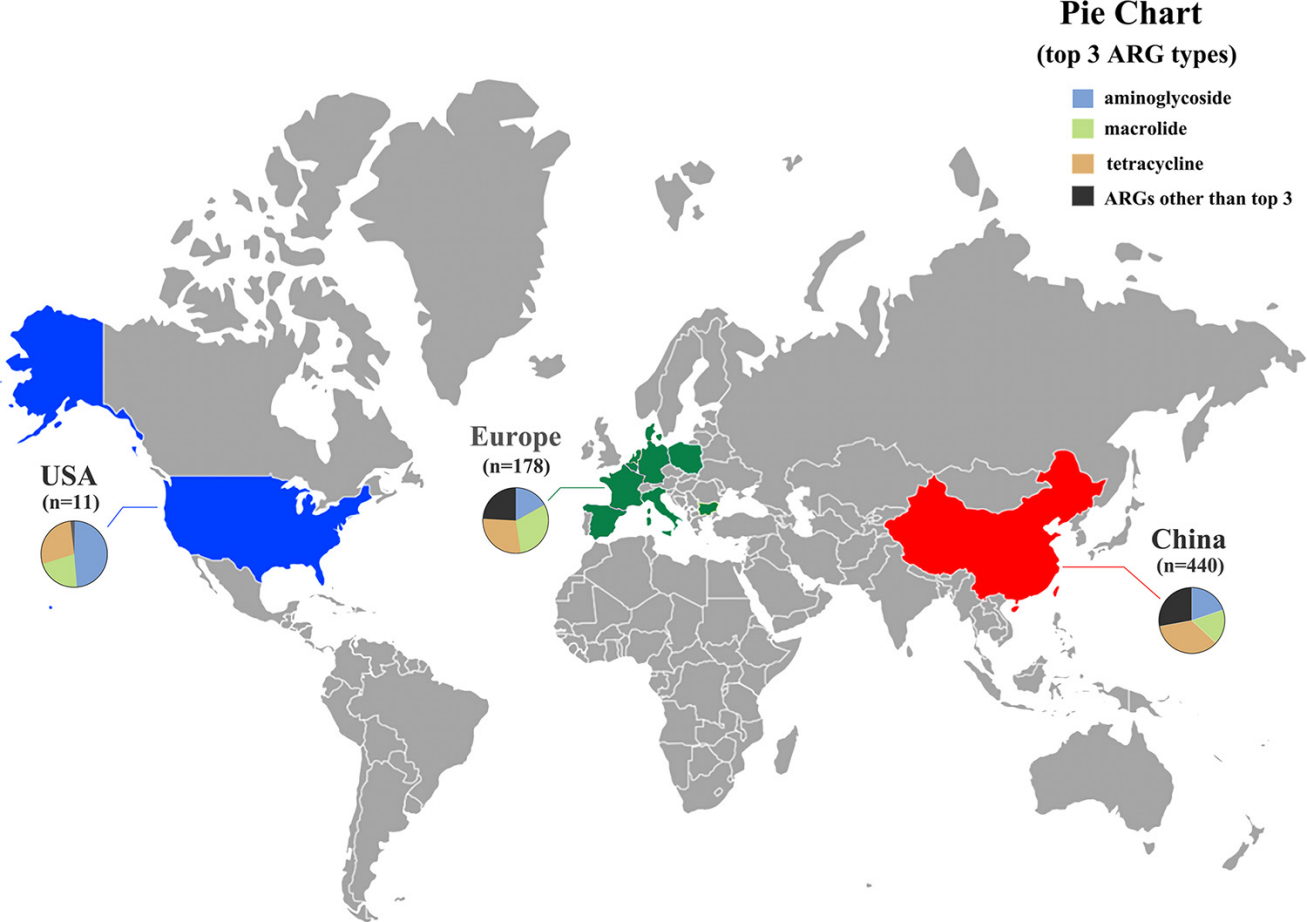
Distribution map of chicken gut samples and diversity of ARGs, pie chart showing the profile of ARG abundance (top 3 ARG types). Europe (*n* = 178) includes Netherland s(*n* = 20), Germany (*n* = 19), France (*n* = 20), Spain (*n* = 20), Belgium (*n* = 20), Italy (*n* = 20), Poland (*n* = 20), Denmark (*n* = 20), and Bulgaria (*n* = 19). The map was created using the generator at https://pixelmap.amcharts.com/.

### Quality control and metagenome assembly.

First, raw reads with average quality scores < 20 (Q20) or length < 20 bp (L20) were removed using Sickle (53) (https://github.com/najoshi/sickle). After that, the metagenomic sequencing data of each sample were *de novo* assembled with default k-mer size using the CLC Genomics Workbench (Version 10.0.1, CLC Bio, Aarhus, Denmark), and the assembled contigs longer than 500 bp were reserved. We finally obtained 35672711 contigs from 629 metagenomic samples. The detailed information is summarized in Table S2.

### Identification of ARG-like ORFs and the mobile genetic elements (MGEs).

The open reading frames (ORFs) of the contigs were predicted using Prodigal v2.6.3 with a metamodel (54). The ARG-like ORFs were identified against the resfinder database (55) using BLASTN with an E-value of ≤ 1 × 10^−5^, and the results that identity ≥ 80% and over 70% query coverage were identified as ARG-like ORFs (16). The coverage of ARG-like ORFs was calculated by mapping metagenomic reads to the contigs with a minimum length coverage of 95% at 95% similarity and contigs minimum length ≥ 500bp using the CLC Genomics Workbench (56). The number of cells in each metagenome sample was calculated by ARGs-OAP v2.0 (57) (Table S1 for complete sample cell count information). To compare the coverage of each different sample, the ARG-like ORFs coverage was normalized by the number of cells in each sample (copies/cell). The calculation formula was as follows:

$$Coverage\;=\overset{\text{n}}{\underset{1}{\sum }}\frac{N\times 150/L}{C}$$

where N is the number of reads mapped to ARG-like ORFs, L is the sequence length of the target ARG-like ORFs, n is the number of ARG-like ORFs, and 150 is the length of Illumina sequencing reads (52, 56), and the C means the cell number of per sample.

Likewise, the MGEs database including plasmid, insertion sequences (ISs), and Integron was identified against the plasmidfinder (58), ISfinder (59), and Integron (60) databases, respectively. The final abundance calculation method was the same as the above equation remains the same.

### Phylogenetic analyses.

All phylogenetic trees were inferred using MEGA X (Version 10.1.8) with 1000 bootstraps (61). To construct phylogenetic relationships of important ARGs, the corresponding sequences of *tetX* (*n* = 136), *mcr* (*n* = 22), and *bla*_NDM_ (*n* = 13) were analyzed, separately. The protein of sequence was aligned using clustalw. The phylogenetic tree was plotted with iTol (Version 5.7) (62).

### Identification of bacterial host of ARG-carrying contigs.

The predicted protein sequences of ORFs from the contigs that carried ARG-like ORFs were annotated using BLASTP against non-redundant protein NCBI databases at *E*-value ≤ 1 × 10^−5^ (63). The results were annotated using ETE Toolkit (version 2.3) for the assignment of taxonomic (64). In short, if more than 50% of the ORFs on a contig were attributed to the same kingdom/phylum/class/order/family/genus, then the contig was assigned to that taxon (65).

### Genome reconstruction of gut microbes and genome annotation of MAGs.

With the functional module of metaWRAP (v1.2.1), a pipeline that contains several modules for the study of metagenomic bins (66), genome reconstruction of gut microbes using metagenomic sequencing data is carried out. Briefly, the metagenome-assembled genomes (MAGs) were constructed using contigs by the binning algorithm “MetaBAT2” (67) in the metaWRAP software. The default of the minimum length of contigs used for constructing bins with MetaBAT2 was 1500 bp.

CheckM v1.1.2 with options lineage_wf, -x fa was used to assess the completeness and contamination of all MAGs (20). After filtering for completeness ≥ 80% and contamination ≤ 10% (68), we were left with 4062 high-quality bins from the 629 samples. These high-quality bins were annotated to the genus using the Genome Taxonomy Database Toolkit (GTDB-Tk) (69).

### Statistical analysis.

The heatmap, correlation heatmap, and variance analysis were generated using the pheatmap, ggcorrplo, and limma packages in the R package (v4.0.2, https://www.r-project.org/), respectively. The alpha diversity of the gut microbiota, including the number of species and the Shannon index, was calculated by spaa and vagen in the R package (v4.0.2). MetaPhlan2 (v2.7.5) (70) was used to identify species and to determine their relative abundances across samples. The network was explored and visualized using the interactive platform of Gephi (v0.9.2). The drawing of simple graphs was done by GraphPad Prism 8. Statistical analyses were performed using SPSS software (PASW Statistics 22.0). The Pearson correlation coefficient was used to show the correlation between the abundance of ARGs and the abundance of MGEs. A two-tailed Wilcoxon test (two-by-two comparison) was used to compare Chinese and European chickens. A *P*-value < 0.05 was considered a statistically significant difference. The Procrustes transformation was performed using two Bray-Curtis distance plots (PCoA) as input based on microbial community matrices and ARGs at the subtype level. R (v4.0.2), Python (v3.7.3) programs were used to handle the text processing, information extraction, and data statistics for this study.

**Data availability.** The data set supporting the conclusions of this article is included within its supplementary file. The 4062 high-quality metagenome-assembled genomes obtained in this study are available in national center for biotechnology information (NCBI, accession No. PRJNA792464). Any remaining information can be obtained from the corresponding author upon reasonable request.
