# Supplementary material for: Metagenomic Insights into Chicken Gut Antibiotic Resistomes and Microbiomes
Source: Microbiol Spectr. 2022 Mar 1;10(2):e01907-21. doi: 10.1128/spectrum.01907-21 (PMC9045286; doi:10.1128/spectrum.01907-21)
Supplement: SUPPLEMENTAL FILE 2 — Supplemental material. Download SPECTRUM01907-21_Supp_2_seq12.pdf, PDF file, 0.9 MB [file spectrum01907-21_supp_2_seq12.pdf]

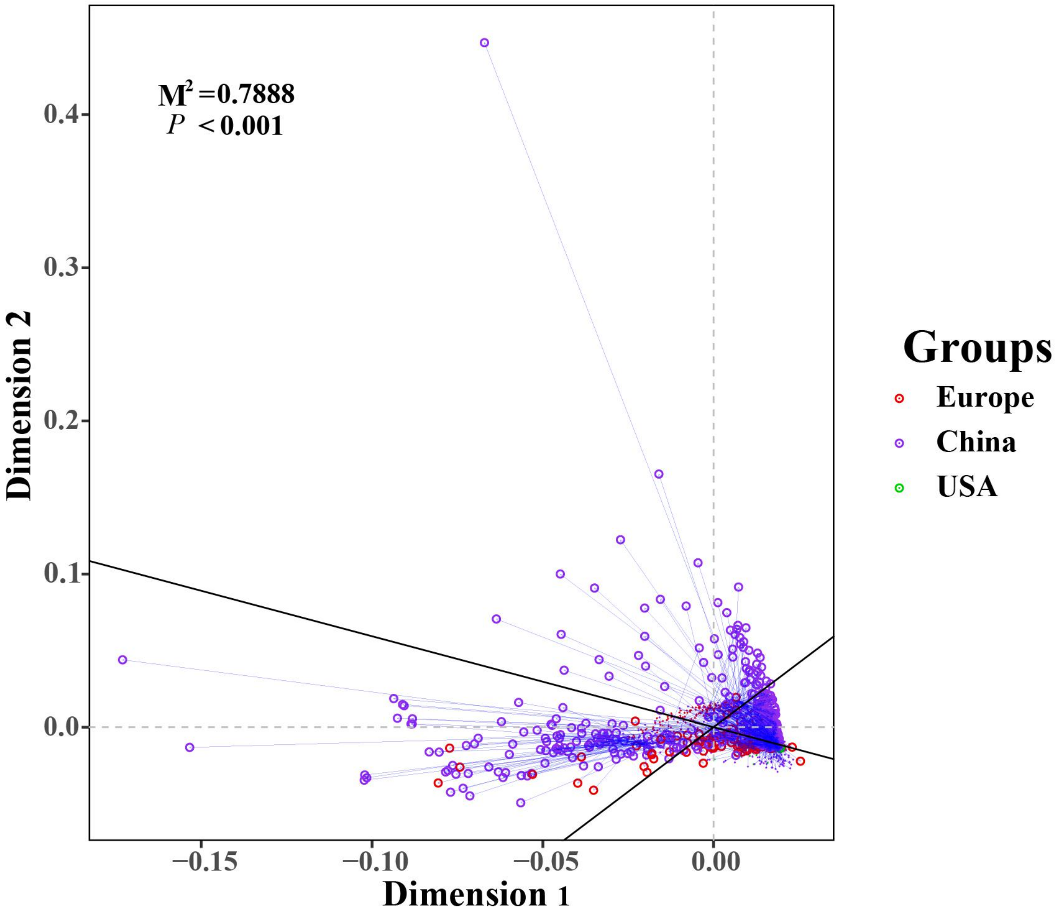

Fig. S1 Procrustes analysis depicts the correlation between ARG content (Bray-Curtis) and bacterial community (Bray-Curtis), M2 = 0.7888, P < 0.001.

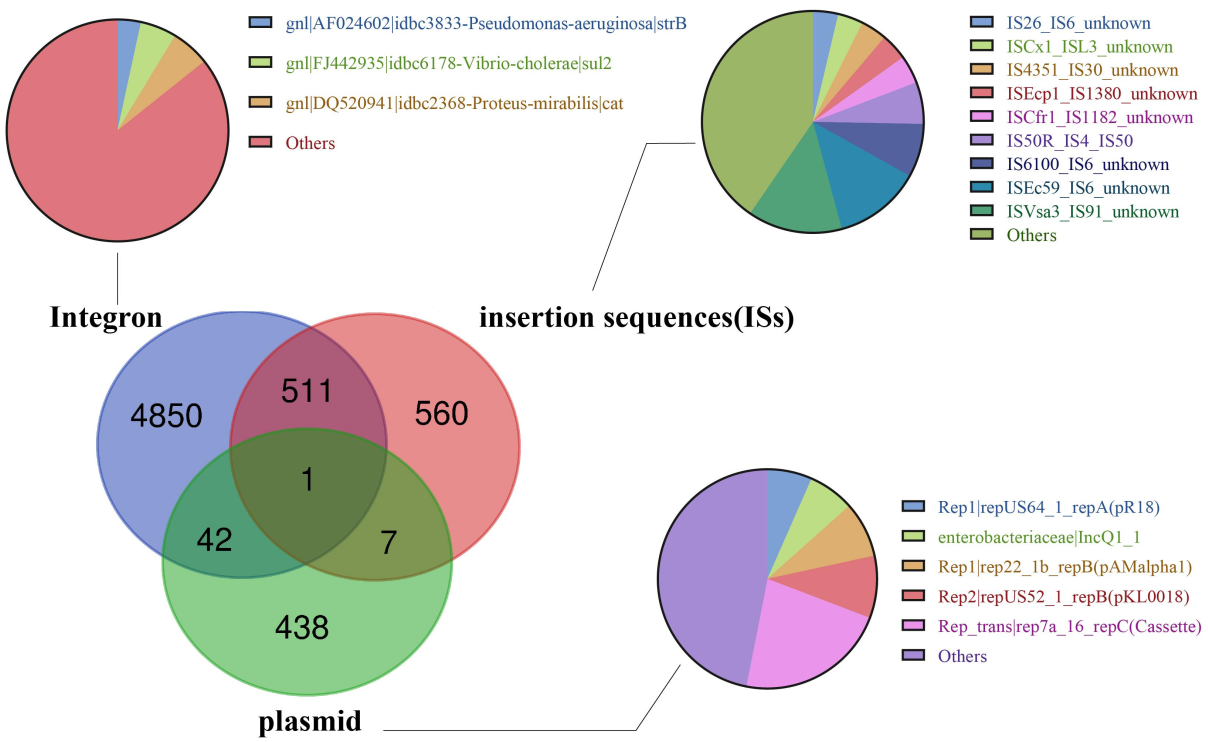

Fig. S2 The Venn diagram shows the distribution of different types of MGEs(Integron, insertion sequence and plasmid) in 6409 contigs with coexisting ARGs and MGEs and shows their major share in each type in a pie chart.

a

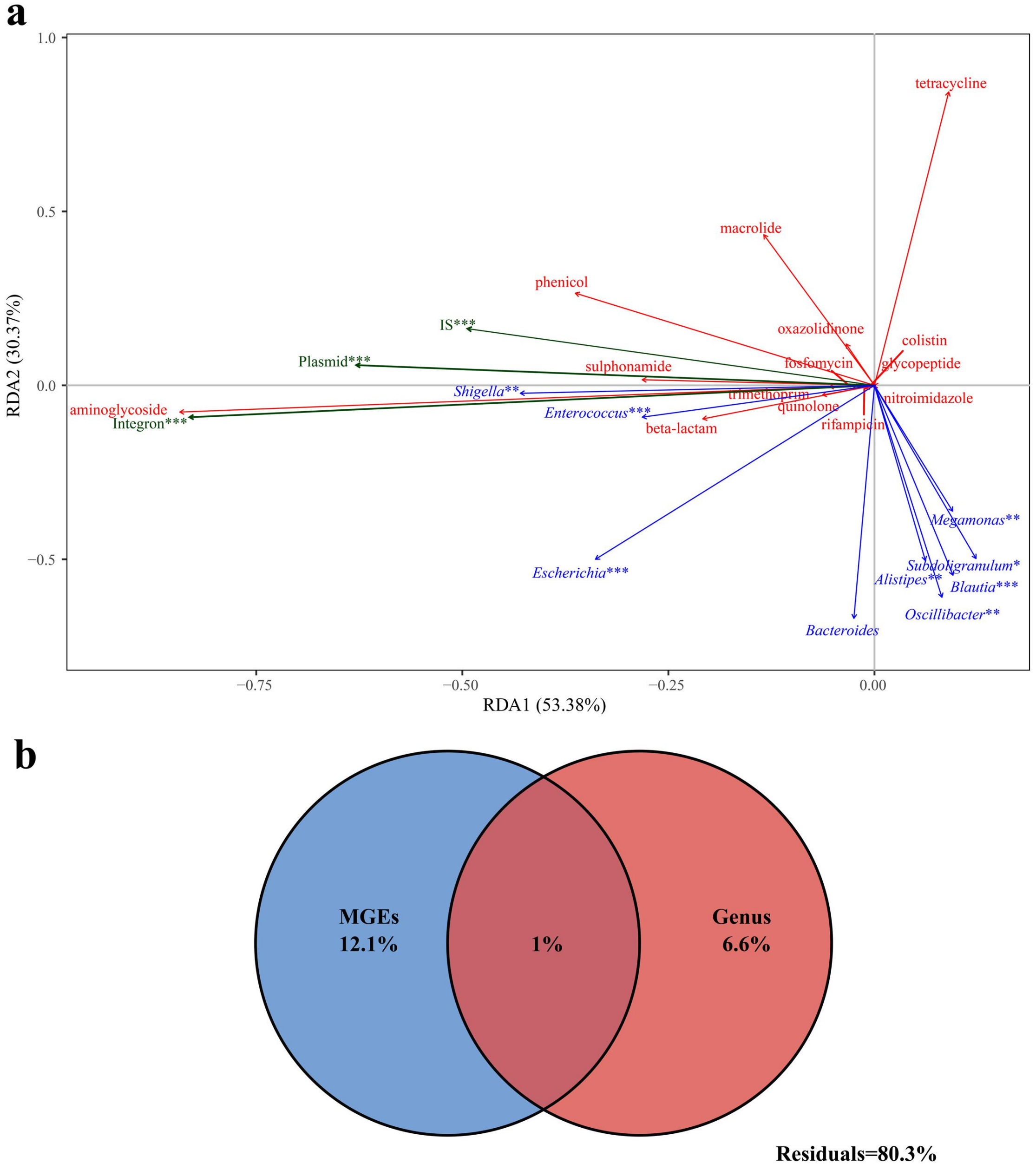

b

Fig. S3 (a) RDA plot reveal the relationship between MGEs and major Genus on ARGs. All of the variables in the model were evaluated for collinearity using the variance inflation factor. $***P < 0.001$, $**P < 0.01$, $*P < 0.05$ (b) Variation partitioning analysis of the effects of MGEs and major Genus on the ARGs.

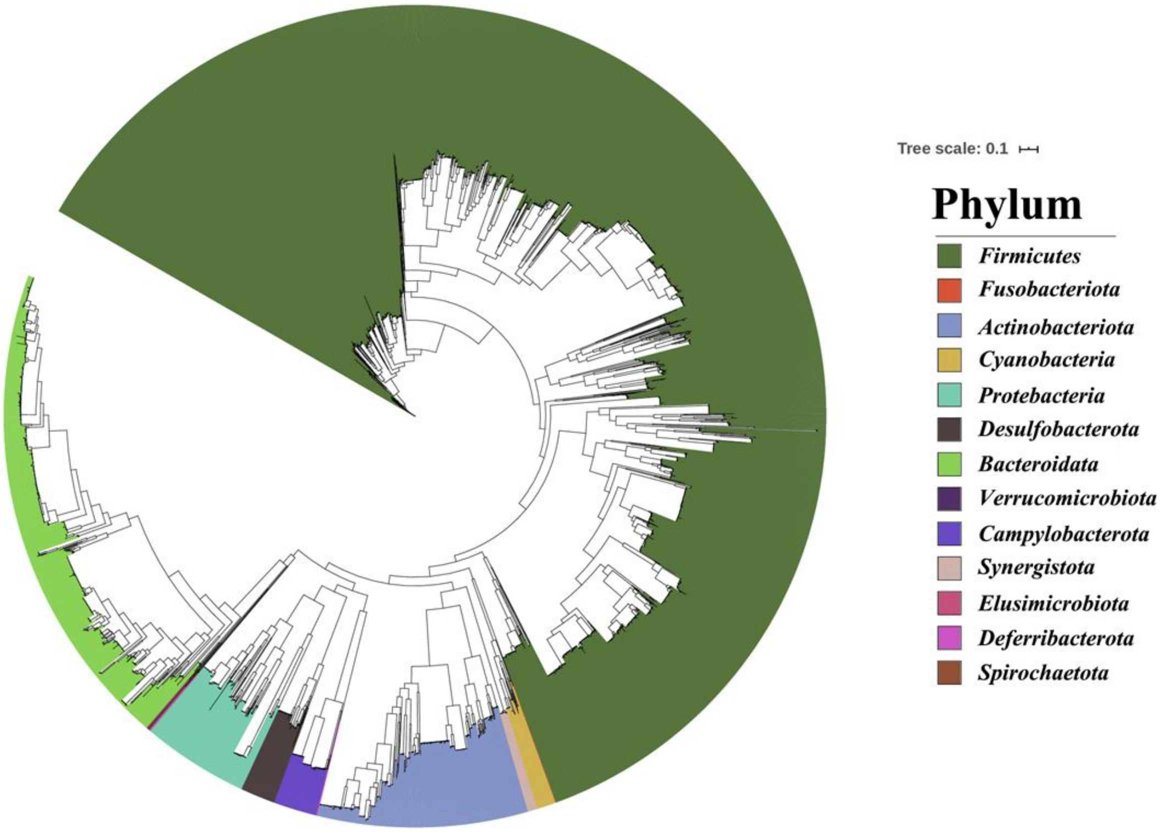

Fig. S4 Phylogenetic tree of 4058 (excluding four archaeal strains) MAGs in the chicken gut, labeled according to the taxonomic order defined by GTDB-TK, with different colors in the branch background representing the phylum.
